# GRAFT MATURATION IN POSTOPERATIVE MAGNETIC RESONANCE IMAGING: A SYSTEMATIC REVIEW AND META-ANALYSIS OF ISOLATED VS. ANTEROLATERAL-AUGMENTED ACL RECONSTRUCTIONS

**DOI:** 10.1590/1413-785220263406e307712

**Published:** 2026-11-09

**Authors:** Arthur Canhada de Albuquerque, Rodrigo Bernstein Conde, Andre Giardino Moreira da Silva, Riccardo Gomes Gobbi, Camilo Partezani Helito, Daniel Peixoto Leal

**Affiliations:** 1 Universidade Federal de Ciencias da Saude de Porto Alegre, Programa de Pos-graduacao em Ciencias da Saude, Porto Alegre, RS, Brazil.; 2 Fundacion H. A. Barcelo, Instituto Universitario de Ciencias de la Salud, Buenos Aires, Argentina.; 3 Universidade de Sao Paulo, Instituto de Ortopedia, Hospital das Clinicas HCFMUSP, Sao Paulo, SP, Brazil.

**Keywords:** Anterior Cruciate Ligament Reconstruction, Tenodesis, Magnetic Resonance Imaging, Signal-To-Noise Ratio, Meta-Analysis, Joint Instability, Reconstrução do Ligamento Cruzado Anterior, Tenodese, Imageamento por Ressonância Magnética, Razão Sinal-Ruído, Metanálise, Instabilidade Articular

## Abstract

**Objectives:**

This study aims to evaluate whether the association of an anterolateral extra-articular procedure (tenodesis or anterolateral ligament reconstruction) to anterior cruciate ligament reconstruction (ACLR) correlates with graft maturation time.

**Methods:**

We systematically searched PubMed, Embase, and the Cochrane Central for studies comparing the graft maturation time between ACLR with anterolateral augmentation (ALA) and isolated ACLR. We assessed graft maturation, via Magnetic Resonance Imaging (MRI) using the signal-to-noise quotient (SNQ). Statistical analyses were performed using Review Manager 5.4. Odds ratios (OR) and mean differences (MD) with 95% confidence intervals (CIs) were pooled using random-effects models based on heterogeneity (I^2^).

**Results:**

Five studies, including 450 patients, assessed graft maturation using SNQ values. No significant difference was observed between ACLR combined with ALA and isolated ACLR (MD -0.77; 95% CI -3.56–2.03; p=0.59; I^2^=92%), with substantial heterogeneity.

**Conclusions:**

The addition of ALA to ACLR does not appear to significantly enhance graft maturation. While some studies report improved stability, these benefits do not consistently translate into better ligamentization. The decision to augment ACLR should be individualized, considering population and individual characteristics. **Level of Evidence III; Systematic Review**.

## RESUMO

**Objetivos:**

Avaliar se a associação de um procedimento extra-articular anterolateral (tenodese ou reconstrução do ligamento anterolateral) à reconstrução do ligamento cruzado anterior (ACLR) está correlacionada ao tempo de maturação do enxerto.

**Métodos:**

Foi realizada uma busca sistemática no PubMed, Embase e Cochrane Central por estudos comparando o tempo de maturação do enxerto entre ACLR com reforço anterolateral (ALA) e ACLR isolada. A maturação do enxerto foi avaliada por meio de Ressonância Magnética (MRI) utilizando o quociente sinal-ruído (SNQ). As análises estatísticas foram realizadas no Review Manager 5.4. Odds ratios (OR) e diferenças de médias (MD) com intervalos de confiança de 95% (CIs) foram agrupados utilizando modelos de efeitos aleatórios baseados na heterogeneidade (I^2^).

**Resultados:**

Cinco estudos, totalizando 450 pacientes, avaliaram a maturação do enxerto por meio dos valores de SNQ. Não foi observada diferença significativa entre ACLR combinada com ALA e ACLR isolada (MD -0.77; 95% CI -3.56–2.03; p=0.59; I^2^=92%), com heterogeneidade substancial.

**Conclusões:**

A adição de ALA à ACLR não parece melhorar significativamente a maturação do enxerto. Embora alguns estudos relatem melhora da estabilidade, esses benefícios não se traduzem consistentemente em melhor ligamentização. A decisão de associar o reforço à ACLR deve ser individualizada, considerando as características da população estudada e de cada paciente. ***Nível de Evidência III; Revisão Sistemática***.

## INTRODUCTION

The anterior cruciate ligament (ACL) is a critical structure in knee biomechanics, primarily responsible for restraining anterior tibial translation and maintaining rotational stability.^1^ ACL injuries are among the most common knee injuries, particularly in athletes engaged in pivoting sports, often leading to functional instability and impairing daily activities and sports participation.^2^ The gold-standard treatment is arthroscopic anterior cruciate ligament reconstruction (ACLR) using autografts, such as hamstring or patellar tendon grafts.^3^ Following reconstruction, these grafts undergo a complex biological process known as ligamentization, where tendon tissue gradually remodels into a structure resembling the native ACL in morphology and mechanical properties.^4,5^ Factors interfering with this process can increase failure risk, manifesting as residual instability or re-rupture. Proper rehabilitation during this vulnerable phase is essential for successful incorporation.^6^

While ACLR is the standard treatment for ACL deficiency, failure rates remain a concern.^7-10^ Primary ACLR failure ranges from 3.2% to 11.1%, reaching 34.2% in high-risk populations, such as young athletes.^2,11^ Furthermore, up to 25% of reconstructions result in residual pivot shift, indicating that this technique alone may be insufficient to fully restore knee kinematics.^12,13^ Consequently, the role of the anterolateral complex (ALC) has gained attention. Defined as secondary rotational stabilizers, these structures, such as the anterolateral ligament (ALL), are believed to contribute to pivot shift in ACL-deficient knees and have been proposed as potential adjuncts to ACLR for improving knee stability and possibly influencing graft maturation.^1,9,12,13^ Biomechanically, anterolateral procedures aim to limit internal rotation and reduce graft strain, potentially enhancing ligamentization and decreasing failure rates.

Given these hypotheses, anterolateral augmentation is increasingly considered for high-risk populations (young, athletes, females, or those with explosive pivot-shift).^6,9,13,14^ Combining ACLR with anterolateral augmentation (ALA), like lateral extra-articular tenodesis (LET) or anterolateral ligament reconstruction (ALLR), could reduce residual pivot shift and improve outcomes.^3,8,9,15^ However, evidence regarding the impact of these additional procedures on graft maturation remains inconsistent. While some studies suggest improved remodeling with combined techniques^16-18^, others report no significant difference in ligamentization compared to isolated ACLR.^11,19^

These conflicting findings highlight the need for a comprehensive evidence synthesis. Therefore, this meta-analysis aims to systematically evaluate whether the addition of anterolateral extra-articular augmentation to ACLR has some (positive or negative) effect on graft maturation time. By analyzing magnetic resonance imaging (MRI) to assess signal-to-noise quotient (SNQ) values, we seek to clarify the role of these combined procedures in the ligamentization process.

## METHODS

This systematic review and meta-analysis follows the guidelines of the Cochrane Collaboration Handbook for Systematic Reviews of Interventions and the Preferred Reporting Items for Systematic Reviews and Meta-Analyses (PRISMA) Statement.^20,21^ The protocol was prospectively registered at PROSPERO on January 18, 2025 (ID: CRD 42025628492). No amendments were made to the registered protocol.

### Eligibility criteria

Inclusion was restricted to: (1) randomized clinical trials (RCT) or non-RCT or prospective/ retrospective cohorts; (2) comparing ACLR with anterolateral reinforcement to ACLR isolated; and (3) evaluating graft maturation through MRI. We excluded studies with (1) no control group, (2) revision ACLR, (3) multiple ligament injuries, (4) double-bundle ACLR technique and (5) no use of signal-to-noise quotient (SNQ) to evaluate graft ligamentization.

### Search strategy and data extraction

It was systematically searched in PubMed, Embase and Cochrane Central (last searched: January 2025) the following search terms: ‘ACL’, ‘anterior cruciate ligament’, ‘anterolateral ligament’, ‘lateral extra-articular tenodesis’, ‘lateral extra-articular procedure’, ‘LET’, ‘Lemaire’, ‘anterolateral ligament reconstruction’, ‘anterolateral ligament augmentation’, ‘extra-articular augmentation’, ‘lateral tenodesis’, ‘lateral reinforcement’, ‘ALL’, ‘MRI’, ‘magnetic resonance imaging’, ‘nuclear magnetic resonance imaging’. References from all included studies were also manually searched for any additional studies. Two authors independently extracted the data following predefined search criteria and quality assessment. Discrepancies were resolved by a third reviewer. Full search strategies for all databases are available as Supplementary Material.

### Endpoints and subgroup analyses

The primary outcome of interest was graft maturation, measured using SNQ values obtained from postoperative MRI scans. Prespecified subanalyses included data restricted to: (1) femoral tunnel drilling technique; and (2) anterolateral augmentation technique.

#### Endpoint measurement

The primary outcome was evaluated using MRI-based SNQ values, which serve as a parameter of graft structural properties and vascularization. SNQ is calculated by subtracting the signal intensity of a reference tissue from that of the graft and dividing the result by the background noise signal. Higher SNQ values are generally associated with increased water content and vascularity, suggesting lower graft maturity. Weiller et al.^22^ demonstrated that SNQ values inversely correlate with graft tensile strength and are associated with histological alterations and biomechanical changes during ligamentization.

### Quality assessment and publication bias

Two authors independently assessed the quality of the included studies using version 2 of the Cochrane Risk of Bias in Non-randomized Studies – of Interventions (ROBINS-I) assessment tool.^23^ Disagreements were addressed and resolved through a consensus after discussing the reasons for the discrepancy. Publication bias was evaluated by funnel-plot analysis of point estimates about study weights. As per Cochrane's recommendations, Egger's test was not performed due to the number of included studies in this meta-analysis (n < 10).^20^ Certainty of evidence was not formally assessed using GRADE due to the limited number of included studies (n=5) and the observational nature of all included studies.

### Statistical analysis

Odds ratios (OR) with 95% confidence intervals were used to compare treatment effects for categorical outcomes, while mean differences (MD) were applied to continuous variables. Heterogeneity was classified as low when I^2^ < 25% and moderate to high when I^2^ ≥ 25%. The DerSimonian-Laird random-effects model was applied when I^2^ ≥ 25%.. No data conversions or imputations were required. A sensitivity analysis was performed using the leave-one-out approach. All statistical analyses were conducted using Review Manager 5.4 (Nordic Cochrane Centre, The Cochrane Collaboration, Copenhagen, Denmark). Results are displayed as forest plots, a risk of bias summary and a funnel plot.

## RESULTS

### Study selection and characteristics

The initial search identified 635 studies. After excluding duplicates and unrelated articles, 19 underwent a thorough review, with 5 meeting the final criteria for qualitative and quantitative analysis (Figure 1). The analysis included data from 450 patients across 5 retrospective cohort studies, of whom 187 (41.5%) underwent ALCR + ALA. Population characteristics are presented in Table 1. The studies were heterogeneous regarding graft type, surgical technique, follow-up to MRI period and baseline characteristics. We performed a subgroup analysis focusing on similarities to mitigate potential bias.

**Figure 1:**
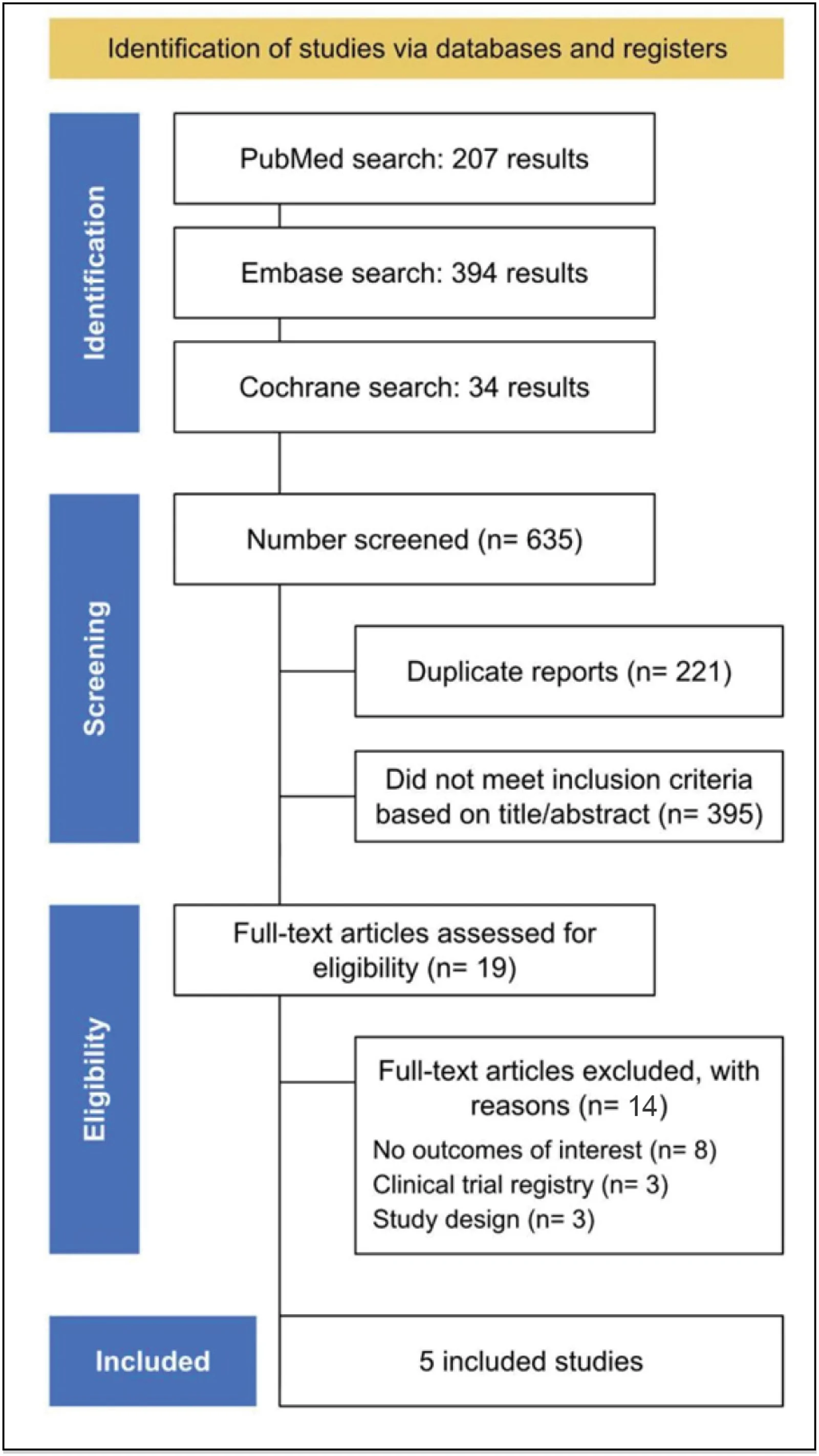
PRISMA flow diagram of study screening and selection.

The flowchart illustrates the stages of identification, screening, eligibility, and inclusion. From an initial search yielding 635 records across PubMed, Embase, and Cochrane databases, 221 duplicates were removed. After screening titles and abstracts, 19 full-text articles were assessed for eligibility, resulting in a final inclusion of 5 studies for qualitative and quantitative synthesis.

**Table 1:** Baseline characteristics of included studies.

Study	Country	Study Design	Patients G1/G2	Male (%) G1/G2	Age (years)¶ G1/G2	BMI¶ G1/G2	ALA Type	Graft Type [nº of strands reported]	ACLR Femoral Drilling G1/G2	ACLR Fixation		ALA Fixation		MRI		SNQ Measure [Ref]	Follow-upto MRI(mo)†
										Femoral	Tibial	Femoral	Tibial	Machine	Strength		
Cavaignac et al. (2020)	France	Cohort	31/31	100/ 100	27.2±6.7/ 33.1±8.3	25.2±4.4/ 24.4±3.4	LET	G1 ACLR: ST [4] ALLR: G [2] G2 ACLR: ST [4]	Outside-in/ Outside-in	TightRope (Arthrex)	BIS (Arthrex)	Suture anchor (ConMed)	Spiked staple (Arthrex)	Magnetom Skyra; Siemens 15-channel volume array coil	3.0-T	The mean of 3 samples§ [PCL]	12
Jacob et al. (2024)	Australia	Cohort	40/40	57.5/ 57.5	18.7±3.8/ 18.4±3.6	21.9±8.4/ 24.2±3.5	LEAT	G1 ACLR: ST [4] LEAT: ITB [NR] G2 ACLR: ST [4]	AM portal/ AM portal	TightRope (Arthrex)	TightRope (Arthrex)	PEEK SwiveLock (Arthrex)	NR	Magnetom Skyra; Siemens 15-channel volume array coil	3.0-T	The mean of 3 samples§ [PCL]	12
Rojas et al. (2021)	Spain	Cohort	22/30	45.4/ 43.3	31.1±6.1/ 29.7±8.2	NR	MLLET	G1 ACLR: ST+G [3+3] MLLET: ITB [NR] G2 ACLR: ST+G [4+3]	AM portal/ AM portal	Endobutton	BIS	BIS	NR	Signa HDxt; GE Healthcare 4-channel knee coil	3.0-T	The mean of 3 samples§ [PCL]	10
Yang et al. (2024)	South Korea	Cohort	54/122	81.5/ 82.0	26.7±10.6/ 28.7±12.0	25.6±3.9/ 25.6±4.1	ALLR	G1 ACLR: HS‡ [4] ALLR: TA [NR] G2 ACLR: HS‡ [4]	Outside-in/ Outside-in	TightRope (Arthrex)	BIS	BIS	BIS	Magnetom Skyra; Siemens 15-channel volume array coil	NR	The mean of 3 samples§ [PCL]	35
Yau et al. (2024)	China	Cohort	40/40	100/ 100	26.2±6.0/ 27.0±6.3	24.8±3.4/ 24.4±3.2	ALLR	G1 ACLR: ST+G [2+2] ALLR: ITB [2] (n=14), ST [1] (n=26) G2 ACLR: ST+G [2+2]	AM portal (n=14) and outside-in (n=26)/ AM portal	Cortical. button. (various)	BIS	Suture anchor (Arthrex)	NR	NR	1.5-T	The mean signal of the whole graft [QT]	16

Abbreviations: ACLR = anterior cruciate ligament reconstruction; ALA = anterolateral augmentation; ALLR = anterolateral ligament reconstruction; AM = anteromedial portal; BIS = bioabsorbable interference screws; HS = Hamstring; ITB =. iliotibial band; G = gracilis; G1 = ACLR + anterolateral augmentation group; G2 = Isolated ACLR group; LET = lateral extra-articular tenodesis; LEAT = modified deep Lemaire anterolateral extra-articular tenodesis; MLLET = modified Lemaire anterolateral extra-articular tenodesis; MRI = magnetic resonance imaging; PCL = posterior cruciate ligament; QT = Quadriceps tendon; SNQ = signal-to-noise quotient; ST = semitendineous; T= Tesla; TA = separate fresh frozen tibialis anterior allograft; NR = no report.

Notes:

§ = The samples were taken at a proximal, middle, and distal intra-articular portion of the graft;

¶ = Mean ± standard deviation;

† = For both groups;

‡ = Not specified.

### Graft maturation and subgroup analyses

SNQ values indicated that incorporating LET or ALLR into ACLR does not result in a statistically significant difference in graft maturation (MD -0.77; 95% CI -3.56–2.03; *p*=0.59; I^2^=92%; Figure 2). However, significant heterogeneity suggests variability in the true effect. Sensitivity analysis (leave-one-out) maintained high heterogeneity across all scenarios, with no meaningful change in the overall effect estimate.

**Figure 2:**
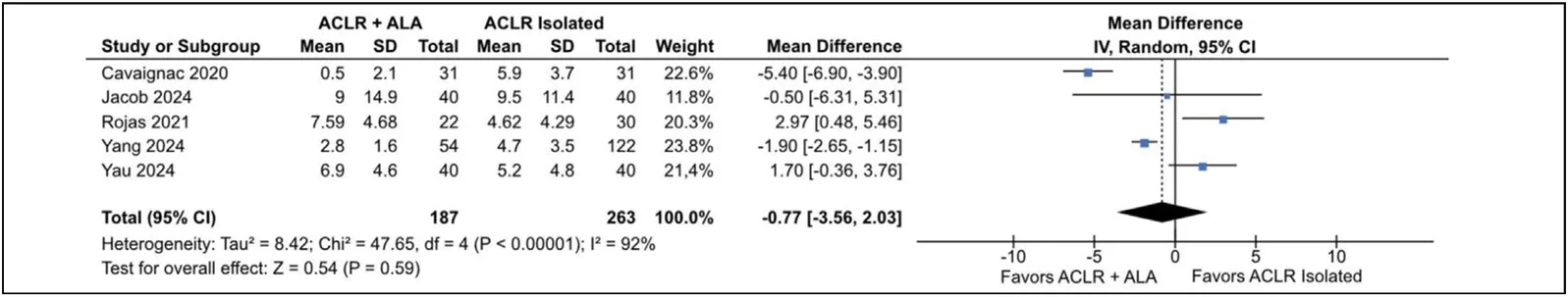
Forest plot of the comparison between ACLR + ALA vs. ACLR Isolated for the primary outcome.

No significant difference in graft maturation between isolated ACLR and ACLR with LET/ALLR (p=0.59), considering a substantial heterogeneity between studies (I^2^=92%). CI = confidence interval; MD = mean difference; IV = inverse variance; ACLR = anterior cruciate ligament reconstruction; ALA = anterolateral augmentation.

Subgroup analysis by anterolateral augmentation technique did not mitigate the observed variability. Both the ALLR group (MD -0.23; 95% CI -3.75–3.29; p=0.90; I^2^=90%; Figure 3a) and the LET group (MD -1.06; 95% CI -7.42–5.29; *p*=0.74; I^2^=94%; Figure 3b) exhibited very high heterogeneity. In contrast, subgroup analysis by femoral drilling technique revealed that the outside-in technique (MD -3.59; 95% CI -7.02– -0.16; p=0.04; I^2^=94%; Figure 4a) indicated a trend favoring the combined procedure, whereas the anteromedial portal approach (MD 3.59; 95% CI 0.50–3.56; p=0.009; I^2^=0%; Figure 4b) suggested a modest benefit for isolated ACLR.

**Figure 3:**
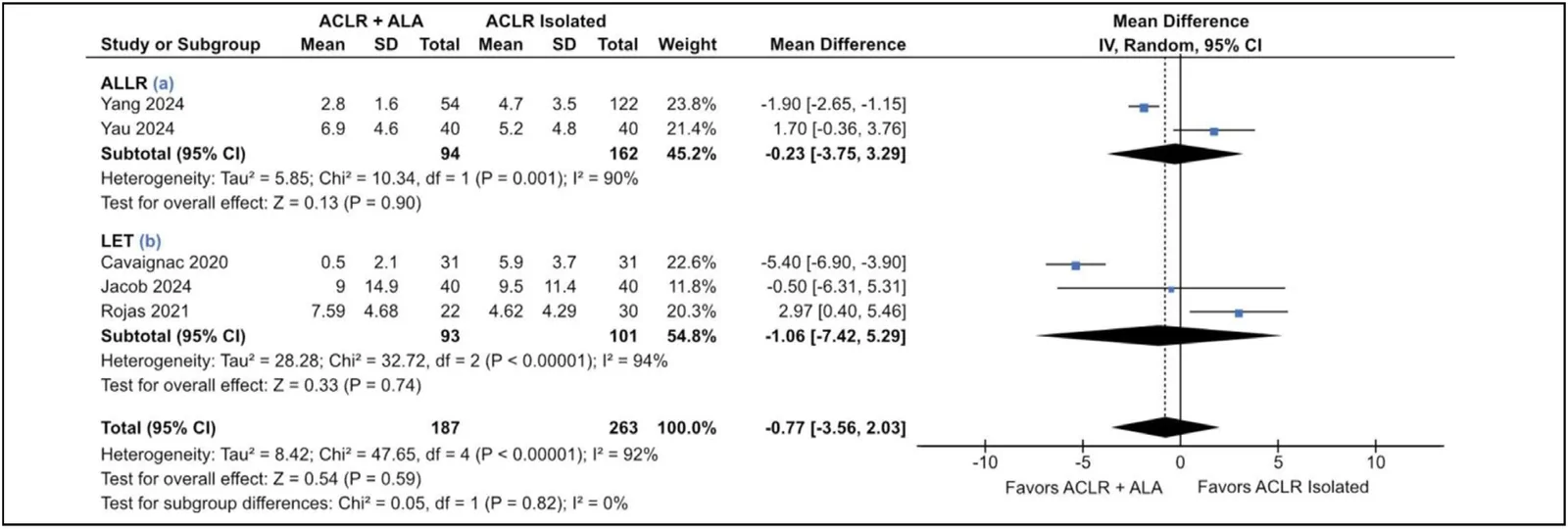
Subgroup analysis based on the type of lateral procedure (LET vs. ALLR).

(a) – ALLR approach favoring ACLR with ALA (p=0.23), despite important heterogeneity (I^2^=94%). (b) – LET technique with no statistically significant difference, with substantial heterogeneity (I^2^=90%). CI = confidence interval; MD = mean difference; IV = inverse variance; ACLR = anterior cruciate ligament reconstruction; ALA = anterolateral augmentation; ALLR = anterolateral ligament reconstruction; LET = lateral extra-articular tenodesis.

**Figure 4:**
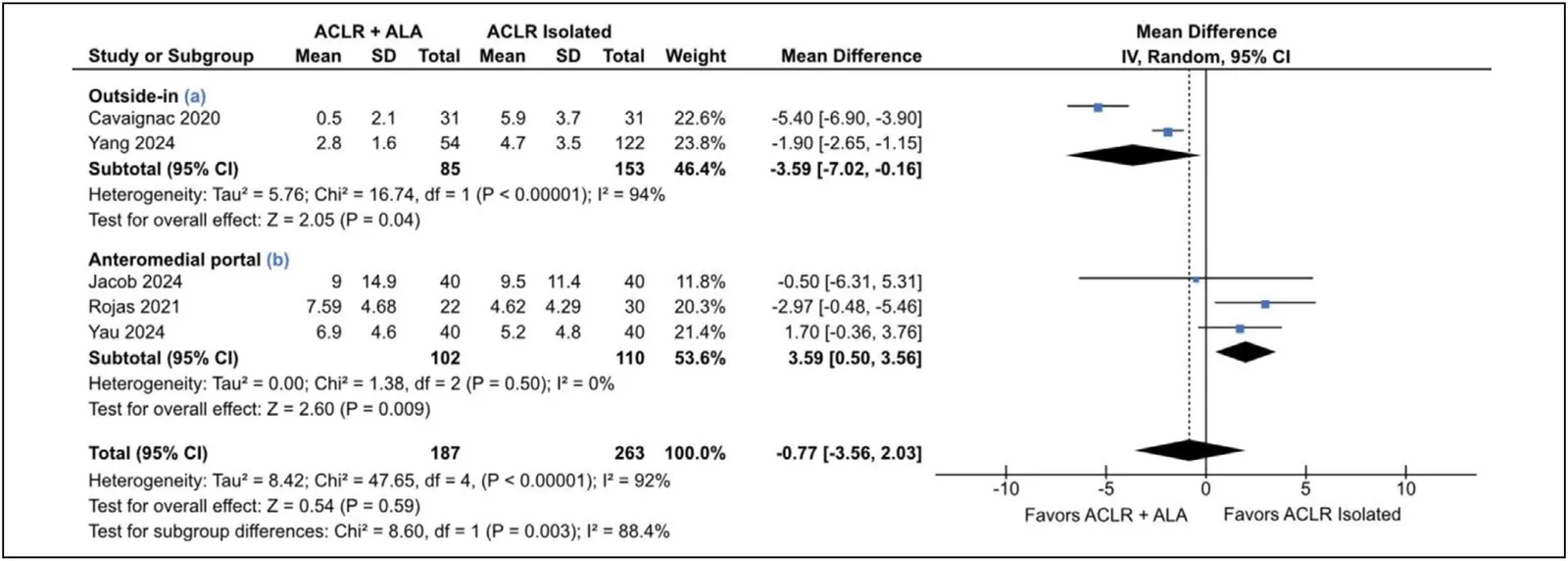
Comparison of Femoral Drilling Techniques in ACLR + ALA: Outside-in vs. Anteromedial Portal.

(a) – Outside-in technique suggesting a modest benefit for ACLR with ALLR (p=0.04), despite important heterogeneity (I^2^=94%). (b) – Anteromedial portal approach favoring isolated ACLR (p=0.009), with low heterogeneity (I^2^=0%). CI = confidence interval; MD = mean difference; IV = inverse variance; ACLR = anterior cruciate ligament reconstruction; ALA = anterolateral augmentation.

### Quality assessment and publication bias

Three of the five included non-randomized studies were identified as having a serious risk of bias due to concerns on the “confounding” domain. The remaining two studies were considered to have a moderate risk of bias, primarily from potential issues related to “confounding”, “participant selection”, and “selection of the reported result”. The risk of bias for each study was assessed using the ROBINS-I tool (Figure 5).^23^ Funnel plot asymmetry (Figure 6) suggested publication bias, indicating that studies with significant results might be more likely to be published than those with null findings. This potential bias is acknowledged as a limitation that may affect the overall interpretation of the results.

**Figure 5:**
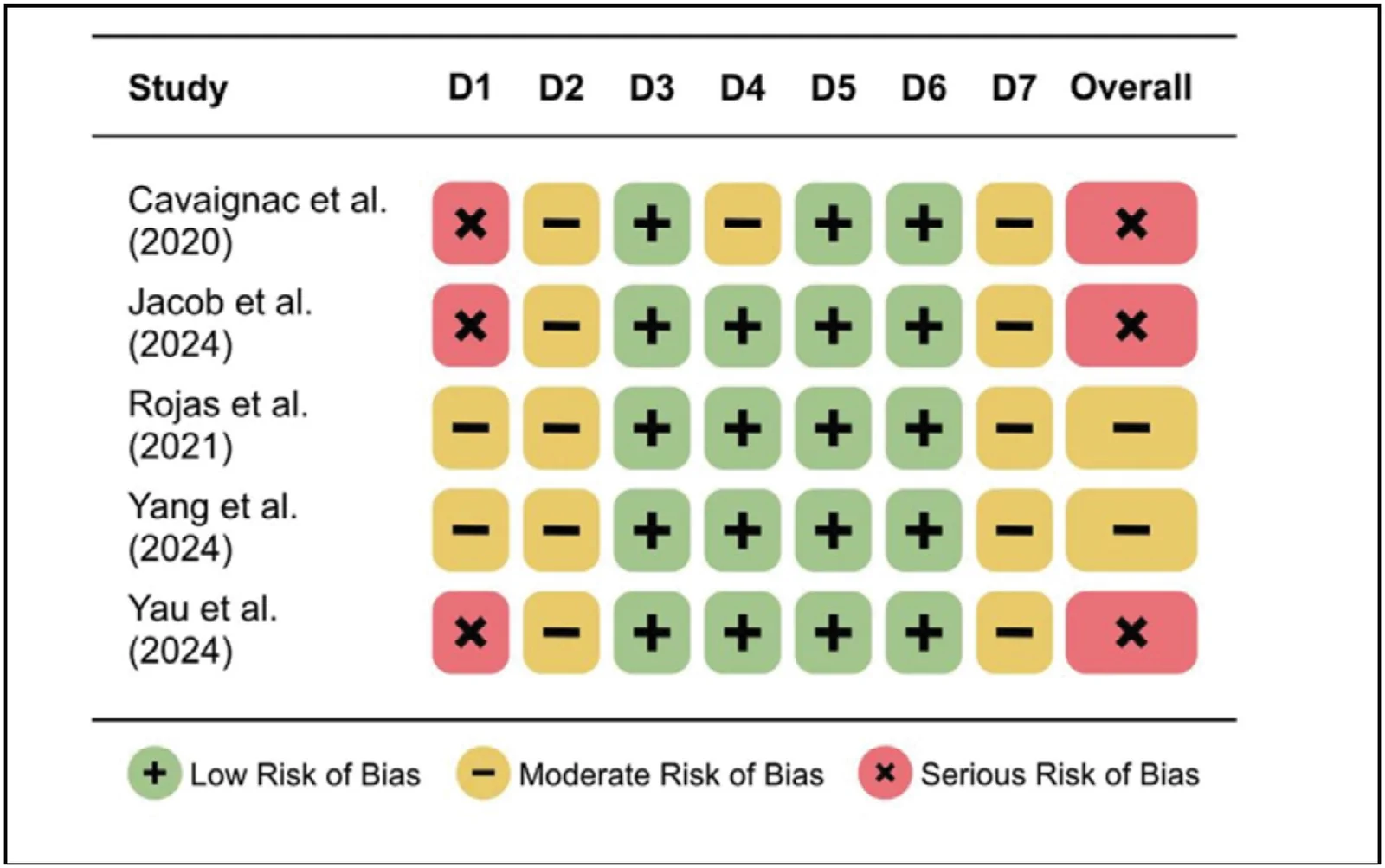
Risk of bias assessment of the included studies.

The traffic light plot illustrates the risk of bias for each study across seven domains: D1, confounding; D2, selection of participants; D3, classification of interventions; D4, deviations from intended interventions; D5, missing data; D6, measurement of outcomes; and D7, selection of the reported result. The symbols represent low risk of bias (+), some concerns (–), and high risk of bias (X). The overall assessment indicates that the majority of studies presented high risk or significant concerns in specific domains, particularly in the D1, D2, and D7 categories.

**Figure 6:**
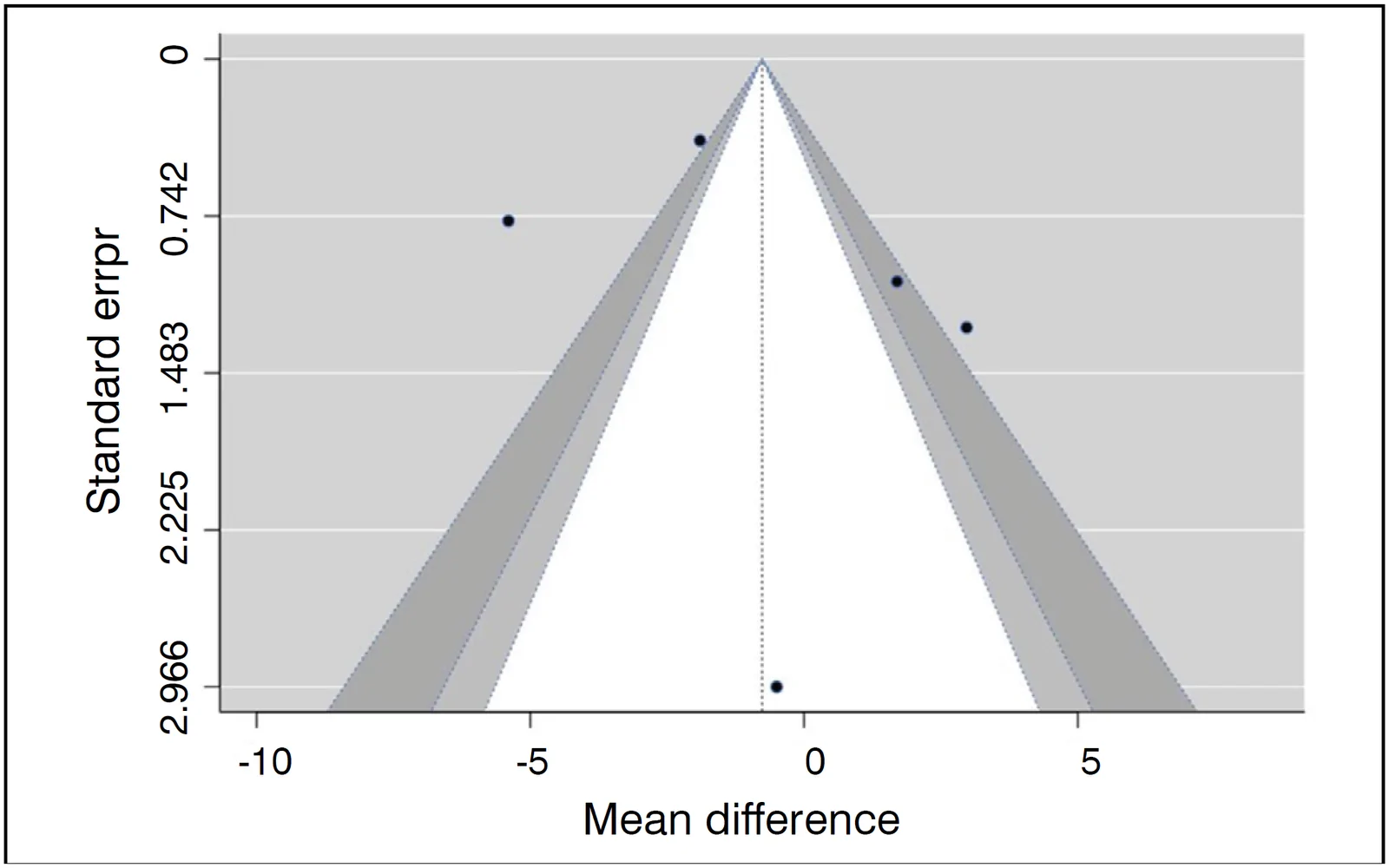
Funnel plot for the assessment of publication bias.

Funnel plot of the mean differences comparing the SNQ values of ACLR isolated and ACLR with ALA. The asymmetric shape of the plot suggests the presence of publication bias. ACLR = anterior cruciate ligament reconstruction; ALA = anterolateral augmentation.

## DISCUSSION

This meta-analysis of five studies evaluating 450 patients found that adding lateral extra-articular augmentation to ACLR did not result in a statistically significant difference in imaging-based ligamentization. While the anteromedial portal technique for femoral drilling suggested a modest favorable effect on maturation for isolated ACLR, the overall results contrast with other meta-analyses, such as de Lima et al.^8^ and Beckers et al.^9^, which reported clear clinical benefits for ALA in primary ACLR.

Beckers et al.^9^ noted that combining primary ACLR with ALA significantly reduces graft rupture risk and improves rotatory stability. These objective improvements in stability do not appear to correlate consistently with enhanced graft maturation via SNQ in our analysis. This suggests that the clinical benefits of ALA might be driven by biomechanical protection rather than accelerated biological ligamentization. Therefore, rehabilitation and return-to-sport timelines should not be modified solely due to an anterolateral procedure.

Similarly, de Lima et al.^8^ found that combined ACL and ALA reconstruction achieved better postoperative clinical outcomes, especially in reducing residual pivot shift and re-rupture rates. Their analysis also indicated lower residual positive Lachman tests and better Lysholm scores in the combined group. While de Lima et al.^8^ focused on ALA reconstruction, their findings, alongside Beckers et al.^9^, underscore that reduced failure rates are achievable with lateral procedures. Our results indicate these advantages are not measurable via SNQ values, reinforcing that the biological maturation rate remains independent of the extra-articular stabilization.

The substantial heterogeneity observed (I^2^=92%) mirrors variability noted in clinical scores by other authors, likely stemming from differences in graft choice (semitendinosus, gracilis, or allografts), femoral drilling approaches, and fixation methods.^8,9^ Furthermore, MRI protocols lack standardization regarding SNQ measurement techniques, reference tissues, and timing. Patient demographics, including age and concomitant injuries like meniscal tears, introduce further variability. For instance, Yang et al.^18^ found that medial meniscal injuries were independently associated with higher SNQ values, indicating worse maturation.

Relying on SNQ as a proxy for maturation is a limitation. As an operator-dependent method, SNQ provides signal intensity data but may not fully capture the complex biological processes of ligamentization.^5^ Nevertheless, included studies utilized multiple evaluators and assessed interobserver reliability, predominantly through the Intraclass Correlation Coefficient (ICC). Reported ICC values ranged from 0.667 to 0.92, indicating moderate-to-excellent agreement.^11,16-19^ Despite this rigor, results remain inconsistent. For instance, Cavaignac et al.^16^ suggested better incorporation with LET, while Jacob et al.^17^ reported less tunnel widening but no improvement in graft healing. Yang et al.^18^ showed superior rotational stability and graft remodeling with ALLR, but there was no conclusive superiority in patient-reported outcomes. Yau et al.^19^ also found no significant difference in graft remodeling between both groups.

Conversely, Rojas et al.^11^ suggest that at 10 months, hamstring autografts in anatomic ACLR with LET exhibited signal intensities indicating inferior ligamentization. This raises the possibility that intra-articular maturation may even be delayed when LET is performed. Despite this, addressing anterolateral structures reduces graft rupture rates at 2 years in high-risk populations and reduces tunnel widening, suggesting clinical benefits may not reflect early MRI signal intensity improvements.^7,17^ Additionally, Ferreti et al.^24^ observed no statistical difference between ALLR and LET regarding maturation and functional outcomes. There is no consensus on when MRI signal intensity reflects ‘normal’ healing, presenting a weak correlation with clinical outcomes.^25^

In skeletally immature patients, Retzky et al.^26^ suggested LET is associated with improved maturation in young athletes, supported by Chipman et al.^27^, who linked ALLR to reduced signal intensity in quadriceps autografts. Perelli et al.^28^ showed that modified Lemaire tenodesis improves stability in this population. Additionally, a pediatric study indicated that ligamentization is a prolonged process, occurring primarily between 12 and 24 months.^29^ In contrast, studies excluding skeletally immature patients found no significant differences,^16,19^, emphasizing that skeletal maturity may be a crucial factor when assessing the benefits of combined procedures.

Patient-specific factors like sex, activity level, and comorbidities may also influence outcomes. Although sex was addressed in several studies, its impact on biological graft maturation remains inconclusive. Cavaignac et al.^16^ excluded female participants due to concerns regarding hormonal fluctuations and their potential effect on graft incorporation, while Rojas et al.^11^ included only males, a factor that limits the external validity of their findings. Conversely, studies that maintained a sex balance across cohorts reported no consistent differences in SNQ values, suggesting that sex may not be a primary predictor of imaging-based outcomes.^17-19^ Patient activity level, however, played a significant role in the clinical decision-making process. High-level athletes and younger patients often demonstrate improved clinical outcomes when ALA is associated with ACLR, likely due to the increased rotational demands.^13,30^ Regarding comorbidities, Yang et al.^18^ identified medial meniscal injuries as an independent risk factor for impaired graft maturation, as evidenced by significantly higher SNQ values. This highlights that the intra-articular environment and concomitant injuries may be as decisive for ligamentization as the surgical technique itself.

In summary, while clinical evidence suggests that anterolateral augmentation provides superior rotational stability and reduces re-rupture rates in primary ACLR^8,9^, our meta-analysis focusing on MRI-based SNQ values indicates that the addition of LET or ALLR does not significantly accelerate or enhance biological graft maturation. The observed clinical benefits are likely attributable to the biomechanical reinforcement provided by the extra-articular procedure rather than a direct modulation of the intra-articular ligamentization process. The high heterogeneity regarding surgical approaches, follow-up durations, and MRI protocols underscores the urgent need for standardized assessment procedures to optimize patient selection. Therefore, the decision to perform ALA should remain individualized, targeting specific high-risk populations where mechanical stability is paramount, while acknowledging the lack of consistent evidence for improved graft maturation based on SNQ values.

### Study limitations

This meta-analysis has limitations. Potential publication bias, suggested by funnel plot asymmetry (Figure 6), may overestimate augmentation effects. High heterogeneity among included studies limits definitive conclusions. Discrepancies may be attributed to: (1) surgical variability in graft and fixation; (2) inclusion criteria differences; (3) variations in imaging timing; and (4) diverse rehabilitation protocols. Subgroup analyses were performed to identify heterogeneity sources and contextualize findings. Acknowledging these limitations, this meta-analysis provides valuable insights into the role of LET/ALLR. Future research can provide definitive evidence regarding combined procedures to guide clinical decision-making.

## CONCLUSION

This meta-analysis reveals that adding ALA to ACLR does not significantly enhance graft maturation. While some studies demonstrate improved rotational stability and reduced tunnel widening, these benefits do not consistently translate into earlier ligamentization. The decision to augment ACLR should be individualized. ALA must not modify rehabilitation and return-to-sports timelines, as it lacks a direct relationship with the biological graft maturation process.

## Data Availability

The contents underlying the research are available in the manuscript.
